# Toward a visuospatial developmental account of sequence-space synesthesia

**DOI:** 10.3389/fnhum.2013.00689

**Published:** 2013-10-25

**Authors:** Mark C. Price, David G. Pearson

**Affiliations:** ^1^Psychology Faculty, University of BergenBergen, Norway; ^2^School of Psychology, University of AberdeenAberdeen, UK

**Keywords:** synesthesia, sequence-space synesthesia, spatial form, visuospatial, mental imagery, individual difference, development, working memory

## Abstract

Sequence-space synesthetes experience some sequences (e.g., numbers, calendar units) as arranged in *spatial forms*, i.e., spatial patterns in their mind's eye or even outside their body. Various explanations have been offered for this phenomenon. Here we argue that these spatial forms are continuous with varieties of non-synesthetic visuospatial imagery and share their central characteristics. This includes their dynamic and elaborative nature, their involuntary feel, and consistency over time. Drawing from literatures on mental imagery and working memory, we suggest how the initial acquisition and subsequent elaboration of spatial forms could be accounted for in terms of the known developmental trajectory of visuospatial representations. This extends from the formation of image-based representations of verbal material in childhood to the later maturation of dynamic control of imagery. Individual differences in the development of visuospatial style also account for variation in the character of spatial forms, e.g., in terms of distinctions such as visual versus spatial imagery, or ego-centric versus object-based transformations.

In sequence-space synesthesia, sequences members such as calendar units or numerals are felt to occupy locations within an explicit spatial layout of sequence members – a *spatial form* – that resides in imaginal or extra-bodily space, and ranges from simple 2D to intricate 3D arrangements (Cytowic and Eagleman, 2009; Simner, 2009; Price and Mattingley, 2013; Jonas and Jarick, in press; Price, in press). Typically, the majority of a synesthete’s spatial forms originate in childhood. While understanding the development of non-numerical sequence knowledge is in its infancy (Berteletti et al., 2012), even less is known about the developmental origins of sequence-space synesthesia, despite a common incidence [though population estimates vary widely from 2% (Brang et al., 2010) to 20% (Sagiv et al., 2006)]. Some suggest unusual brain processes are involved, perhaps mediated by exaggerated cortical connectivity (e.g., Hubbard et al., 2005, 2011; Tang et al., 2008; Eagleman, 2009). This view sometimes links spatial forms to normal processes of implicit spatial representation of magnitude (Hubbard et al., 2005, 2011; Tang et al., 2008; Simner, 2009). Others suggest spatial forms are continuous with standard varieties of visuospatial mental imagery and best understood as a residue from childhood strategies for encoding abstract verbal sequences (Galton, 1880, 1881; Phillips, 1897; Seron et al., 1992; Sagiv et al., 2006; Price, 2009, in press; Jonas et al., 2011; Price and Mattingley, 2013).

In the latter vein, we offer here a speculative summary of how initial acquisition and subsequent elaboration of spatial forms could be accounted for in terms of the known developmental trajectory of visuospatial representations, drawing from the interconnected literatures on mental imagery and visuospatial working memory.

## SEQUENCE-SPACE SYNESTHESIA IS A VARIETY OF VISUOSPATIAL IMAGERY

Sequence-space synesthesia is characterized by internally generated visual and/or spatial sensory experience, and has many experiential similarities with non-synesthetic imagery. We therefore suggest that it is *per definition* a variety of visuospatial imagery, and an example of the considerable individual differences in vividness and prevalence of mental imagery among the general population (McKelvie, 1995; Borst and Kosslyn, 2010). Visuospatial imagery occurs in many contexts, both normal and clinical, but its basic neurocognitive substrates are conventionally taken to be the same across domains. On grounds of explanatory parsimony, we therefore also suggest that standard mechanisms for the production and transformation of conscious visuospatial representations are at very least involved in mediating spatial forms, perhaps even sufficient (Price and Mattingley, 2013; Price, in press). Consistent with this, sequence-space synesthetes show above-average (although not exceptional) non-synesthetic visual imagery at a subjective level (Price, 2009; Rizza and Price, 2012; Meier and Rothen, 2013), and possibly also above-average visuospatial skill at a behavioral level (see Price, in press), including the ability to retain other synesthetes’ forms more accurately than non-synesthetes can (Brang et al., 2010). Furthermore, at least some behavioral correlates of sequence-space synesthesia can be mimicked by use of controlled visuospatial imagery in non-synesthete participants (Price, 2009).

## EARLY ACQUISITION OF EXPLICIT SPATIAL FORMS

At a general level the use of spatial representations to help categorize the world is acknowledged as important in children’s learning (e.g., Namy et al., 1997; Schwartz and Heiser, 2006) and the ability to construct abstract spatial representations from verbal symbols is a cornerstone of human spatial cognition (Pazzaglia et al., 2012). For example, there is some evidence for an imagery-based “mental time line” in adult participants where temporal events are spatially mapped (Arzy et al., 2009). Heine et al. (2011) have also presented EEG data from elementary school children indicating visual imagery activity during numerical comparisons.

More specifically, initial learning of the sequential ordering of acquired verbal symbols such as numerals or calendar units will benefit from visuospatial representations because, prior to around 7 years, children tend to show little use of their phonological loop to rehearse verbal sequences in working memory (Flavell et al., 1966; Hitch et al., 1988). Whether spatial forms develop may therefore depend on individual differences in the ages at which a given child learns such sequences relative to maturation of their phonological rehearsal. However, even when phonological rehearsal is available, visuospatial representations will benefit learning and long-term retention due to the documented advantages of dual coding (Paivio, 1971; Clark and Paivio, 1991). Apparent randomness and irregularity in the layout of some spatial forms may partly derive from acquisition at these early ages when executive control over image generation is underdeveloped, and when spatial layout is less constrained by social conventions of, for example, left to right writing or circular clockwise time representation.

Persistence of visuospatial representations into later childhood and adulthood will be influenced by continued individual differences in reliance on verbal versus non-verbal strategies. In this respect it is notable that adult sequence-space synesthetes report prevalent general visual imagery, may show above-average visuospatial skill in some domains, and seem to adopt more visuospatial strategies (e.g., during some types of mental arithmetic; Ward et al., 2009). Seron et al. (1992) also reported below-average scores on self-report measures of verbalizing tendency although this was not replicated by Meier and Rothen (2013).

However it is important to acknowledge that spatial forms probably derive from multiple developmental influences. If they resulted solely from a developmental delay in phonological rehearsal, we would expect sequences that are learned earliest in life to be most prone to elicit spatial forms. But this is not the case. Numbers 1–30 are on average acquired by children before the calendar sequence of 12 months, and can be expressed earlier in a linear spatial layout (see Table 1 in Berteletti et al., 2012). On the other hand, number forms appear to be much less common than month forms, occurring largely in people who also have other types of form such as months (Phillips, 1897; Price et al., 2009). Therefore, although a minority of synesthetes may develop all their spatial forms due to problematic rote verbal rehearsal in childhood, additional factors must be at play among the majority of synesthetes. For example, it is possible that that calendar sequences are more difficult to learn than numbers, and so are more sensitive to deficiencies in verbal rehearsal. Alternatively, the normal tendency for linear spatial coding of numbers to precede and later generalize to other sequences such as months (Berteletti et al., 2012) may be disrupted, perhaps again owing to deficient verbal encoding, leading to atypical visualization strategies.

## FURTHER ELABORATION OF SPATIAL FORMS

Moving beyond the advantages of visuospatial representations for initial acquisition of sequences, calendar sequences may induce spatial forms more often than numbers because they are more likely to be taught diagrammatically in the first place. Although most synesthetes forget the origins of their forms, some do claim their calendar forms were influenced by exposure to diagrammatic representations from school, TV, calendars, etc. In addition to such environmental influences, continuing developmental incentives for spatial forms may include more complex representational roles such as depicting the cyclical nature of calendar sequences via closed circles.

Once established, spatial forms are reported to increase in complexity from childhood to adulthood (Phillips, 1897; Morton, 1936). This complexity can include creative symbolism such as bends in number lines at decade breaks, distortions of date lines to mark personally significant events, and the use of spatial extent in month or weekday forms to represent the personal importance of some time periods (Price and Mattingley, 2013). By contrast to earlier randomness in forms, controlled and complex imagery of this kind, along with improved processing of spatial relations between objects, will be more dependent on attentional executive-based processes (Pearson et al., 1996; Pearson, 2007) which develop markedly from the age of six onward (Gathercole et al., 2004; Chevalier et al., 2013).

As the ability to generate and transform spatial forms matures, they will evolve many characteristics that are continuous with standard controlled visuospatial imagery. These include elaboration of the forms (e.g., from simple spatial trajectories of sequence members into ribbon or tube-like structures), integration of other associated visual imagery into the form, the continued growth of existing forms such as personal time lines, the development of entirely new forms, and the ability to dynamically transform viewpoint such as zooming into the form or navigating within or around the form (Price and Mattingley, 2013; Price, in press). Skill in shifting one’s attentional window on the form may be exploited by those synesthetes who claim to use their calendar forms as associative mnemonic systems such as weekly or yearly diaries, or use historical time lines to encode autobiographical and biographical dates (Simner et al., 2009). In a minority of unusual synesthetes who report a great number of forms, visualization strategies appear to extend beyond classic sequences to provide general classification systems or mnemonics for shoes sizes, the Lord’s Prayer, etc. Just as self-report measures reveal a wide distribution in the vividness of the mental imagery experienced by non-synesthetes, so the salience and vividness of spatial forms seems to run in graded fashion from the most exotic forms, via vague semi-voluntary forms, to no experience of forms at all.

## INVOLUNTARY FEELING AND CONSISTENCY ARE NOT UNIQUE TO SYNESTHETIC IMAGERY

Although the common claim that sequence-space synesthesia is strongly automatic has been challenged (Price and Mattingley, 2013), spatial forms are nevertheless usually reported to have an involuntary feel. Additionally, although spatial forms do appear to evolve over time, their relative stability as long term representations is one of their notable characteristics (Jonas and Jarick, in press). Involuntary feeling and consistency over time have traditionally been taken as central hallmarks of synesthesia. However even these properties of spatial forms are shared by non-synesthetic visuospatial imagery.

In both healthy and clinical populations, imagery experiences commonly result from involuntary as well as deliberate recall processes (Pearson et al., 2013). Regular experience of recurrent involuntary memory is widespread, and occurs for positive and neutral memories as well as negative ones (Berntsen and Rubin, 2008). Pearson et al. (2013) argue that intrusive mental imagery also plays an important role within clinical psychopathology and is associated with many disorders, including post-traumatic stress disorder and social phobia. In addition, according to the Elaborated Intrusion theory of craving (Kavanagh et al., 2005), mental imagery plays a key role during craving for addictive substances and is a hallmark of the phenomenology associated with craving (Andrade et al., 2012). Intrusive imagery may often seem spontaneous, but arises frequently and with involuntary feel merely due to its ease of retrieval when activated by cues via the normal mechanisms of associative retrieval. These cues may be either external (Keane et al., 1991; Carlesimo, 1994) or internal (Pearson, 2012; Pearson et al., 2012; Krans et al., 2013) in nature. Ease of retrieval also accounts for easy voluntary activation of such images. By analogy, spatial forms seem frequently activated in an involuntary manner by relevant cues (e.g., a month name, or thinking generally about the year calendar), as well as easily activated in a more strategic manner (e.g., strategic memorization via associative placement of retrospective or prospective events within a spatial form, illustrated by exceptional date memory among synesthetes with forms for historical time; Simner et al., 2009). Note that Price and Mattingley (2013) suggest voluntary activation of spatial forms plays a critical role in several experimental paradigms which have been claimed previously to demonstrate the automaticity and inflexibility of synesthetes’ spatial associations.

The content of recurrent intrusive imagery is usually very consistent in terms of what is depicted in the image (Engelhard et al., 2011; Schulze et al., 2013). Alongside ease of access, consistency is also a characteristic of the often complex visuospatial imagery that may develop as a mnemonic strategy among non-synesthetes, including professional mnemonists. Importantly, effective mnemonic imagery needs to be consistent and well-established in long-term memory in order to provide a stable framework for encoding of to-be-remembered material (e.g., Paivio, 1971; Maguire et al., 2003).

## INDIVIDUAL DIFFERENCES IN SPATIAL FORMS REFLECT SEPARABLE SUBCOMPONENTS OF VISUOSPATIAL IMAGERY

In the attempt to establish sequence-space synesthesia as a legitimate and defined topic within cognitive neuroscience, the heterogeneity of spatial forms has tended to be overlooked. Spatial forms vary not merely in their vividness, personal importance, complexity, and involuntariness, but also in their spatial frame of reference, in whether they feel like static depictive visual images or like spatial models, and in the types of spatial transformation that can most naturally be applied to them (Price, in press). Aspects of this rich variation can be naturally accounted for in terms of the separable subcomponents of normal visuospatial imagery, and individual differences in the skills mediated by those subcomponents.

For example, the term “visuospatial imagery” blurs the partial separability, at experiential, behavioral, and neural levels, between “visual” versus “spatial” aspects of visuospatial imagery (Mazard et al., 2004; Kosslyn et al., 2007). While incompletely understood, this distinction refers respectively to a more holistic encoding of the visual or “depictive” appearance of imaged entities, versus more explicit representation of the relative spatial positions of objects or object parts (Hegarty, 2010). It is claimed that individuals tend to be either *object visualizers*, favoring a more visual style of imagery that is associated with vivid high resolution imagery and superior performance on visual memory tasks, or *spatial visualizers*, favoring good spatial analysis and dynamic image transformations such as mental rotation (Kozhevnikov et al., 2005, 2010; Blajenkova et al., 2006). A distinction between object visualizers, spatial visualizers, and verbalizers is also claimed in in the development of cognitive styles among 8- to 17-year olds (Blazhenkova et al., 2011).

There is ongoing debate over whether the experiential character of spatial forms and the behavioral skills of sequence-space synesthetes correspond more closely to the traits of object or spatial visualizers (see Price, in press). For example three studies now show that, for self-report measures, it is in the visual rather than spatial domain that people with “spatial” forms show above-average scores (Price, 2009; Rizza and Price, 2012; Meier and Rothen, 2013; see also Eagleman, 2009). However our observation of synesthetes’ descriptions of their experiences is that spatial forms can reflect characteristics of either trait – i.e., an emphasis on visual detail as seen from one egocentrically defined external viewpoint, or on spatial locations of sequence members in a spatial map that can be navigated as an immersive mental model. One solution to this paradox is that a subset of synesthetes with a spatial visualizer trait have not been detected by studies with small samples or averaged data. Another solution is that visual versus spatial imagery are practically interdependent, even if conceptually separable. For example, a spatial model may be more complex and abstract than a visual image, but visual imagery skill may remain important to instantiate particular views of the spatial model (Pazzaglia et al., 2012). Alternatively, a spatial form could start life as a visual image but develop over time into a spatial model. There is a salient parallel here with literature on human spatial navigation: when people construct spatial representations from verbal input, a hierarchical developmental progression is reported from reliance on landmarks (i.e., an initial focus on specific views), to routes (i.e., a progression through landmarks), to a more flexible survey description (i.e., a spatial model; Nori and Guisberti, 2003). Furthermore, adults seem to show preference for one or other of these levels of spatial representation (Pazzaglia et al., 2000) which, like the overlapping distinction between object/spatial-visualizers, may map onto typologies of spatial form.

For those spatial forms which reach the developmental sophistication of a spatial model, their dynamic transformation is another area where individual differences can be informed by fractionation of visuospatial imagery skills. Some synesthetes report object-centered transformation – e.g., as the months go by, their spatial model of the calendar months rotates around or in front of them. Others report ego-centric transformations – e.g., they feel they navigate through, or around, their spatial model. Price (in press) suggests that synesthetes’ preferred mode of transformation may be related to known individual differences in object- versus ego-centric transformation skill which rely on distinct neural processing resources and are partly independent at a behavioral level, as revealed by visuospatial psychometric tests (Zacks and Michelon, 2005). Interestingly, a classic distinction in spatial metaphor for time among the general non-synesthete population is between ego-moving metaphors (observer feels they move along a time line toward the future) and time-moving metaphors (time is felt to move like a river past the observer; Clark, 1973; Lakoff and Johnson, 1980; Gentner et al., 2002). This raises the possibility that spatial forms and non-synesthetes’ spatial metaphors both reflect similar individual differences in aptitude for one or other mode of visuospatial image transformation.

A practical implication of these kinds of individual differences is that visuospatial skills will differ among synesthetes. Unless synesthetes’ individual profiles are carefully aligned, group comparisons of their behavioral skills versus control samples stand in danger of confusion and inconsistency.

## SPATIAL FORMS AND IMPLICIT SPATIAL REPRESENTATION OF MAGNITUDE

Our approach differs from suggestions (Hubbard et al., 2005, 2011; Tang et al., 2008; Simner, 2009) that spatial forms are an abnormally explicit expression of the analog spatial representations which represent magnitude, not just of numbers but across many continuous perceptual dimensions (A Theory of Magnitude, ATOM, Walsh, 2003). As argued by de Hevia et al. (2006), this use of space as a general analog continuous representation for magnitude, exemplified in the concept of the implicit Mental Number Line (MNL), can be contrasted with the ordering of discrete items of information (e.g., symbolic labels) in representational space. Indeed, neuropsychological data show representation of magnitude and ordinality to be dissociated, even though they interact (Berteletti et al., 2012). We suggest that a fundamental problem in attempting to align spatial forms with systems for magnitude representation is that most spatial forms express non-magnitude sequences, such as calendar units or the alphabet, unlike numbers which possess cardinality as well as ordinality. Moreover, in spatial forms, the use of spatial extension to represent personal importance rather than temporal length of certain months illustrates that magnitude representation is not their main function. Rather, they are easily activated, long-term, visuospatial representations of ordinality which aid some individuals to acquire, retrieve, and mentally navigate within abstract sequences, which can be elaborated to symbolize further information, and which may sometimes provide a facilitative template for further associative memories.

## CONCLUSION

Central hallmarks of sequence-space synesthesia, including involuntary feel, consistency over time, dynamic nature, and elaborative imagery, are not unique to this variety of imagery, but shared by everyday visuospatial imagery, intrusive imagery, and mnemonic imagery. Furthermore, a visuospatial developmental perspective illuminates why and when sequence-space synesthesia originates in some people, and why its character varies between synesthetes. We therefore suggest strong continuity between synesthetic spatial forms and normal processes of visuospatial imagery.

Interest in the relationship between mental imagery and synesthesia is by no means confined to sequence-space synesthesia, and there is growing evidence that other varieties of synesthesia are also associated with above-average visual imagery (Meier and Rothen, 2013; Price, in press). However evidence also suggests that predisposition to experience this variety of synesthesia is separable from other types of synesthesia (Novich et al., 2011), and that it is associated with a different grouping of cognitive styles compared, for example, with grapheme-color synesthesia (Meier and Rothen, 2013). The extent to which our current perspective may extend to other varieties of synesthesia therefore remains an open question.

## Conflict of Interest Statement

The authors declare that the research was conducted in the absence of any commercial or financial relationships that could be construed as a potential conflict of interest.
